# Mechanisms of silver nanoparticle toxicity to the coastal marine diatom Chaetoceros curvisetus

**DOI:** 10.1038/s41598-017-11402-x

**Published:** 2017-09-07

**Authors:** Pablo Lodeiro, Thomas J. Browning, Eric P. Achterberg, Aurélie Guillou, Mohammad S. El-Shahawi

**Affiliations:** 1 Ocean and Earth Science, University of Southampton, National Oceanography Centre, European Way, SO14 3ZH, Southampton, UK; 2 GEOMAR Helmholtz Centre for Ocean Research Kiel, Wischhofstraße 1-3, 24148, Kiel, Germany; 3 Department of Chemistry, Faculty of Science, Damietta University, Damietta, Egypt; 4 Present Address: Department of Chemistry, Faculty of Science, King Abdulaziz University, P. O. Box 80203, Jeddah, 21589 Jeddah, Saudi Arabia

## Abstract

Inputs of silver nanoparticles (AgNPs) to marine waters continue to increase yet mechanisms of AgNPs toxicity to marine phytoplankton are still not well resolved. This study reports a series of toxicity experiments on a representative coastal marine diatom species *Chaetoceros curvisetus* using the reference AgNP, NM-300K. Exposure to AgNPs resulted in photosynthetic impairment and loss of diatom biomass in proportion to the supplied AgNP dose. The underlying mechanism of toxicity was explored via comparing biological responses in parallel experiments. Diatom responses to AgNP, free Ag(I) species, and dialysis bag-retained AgNP treatments showed marked similarity, pointing towards a dominant role of Ag(I) species uptake, rather than NPs themselves, in inducing the toxic response. In marked contrast to previous studies, addition of the organic complexing agent cysteine (Cys) alongside Ag only marginally moderated toxicity, implying AgCys^−^ complexes were bioavailable to this diatom species. A preliminary field experiment with a natural phytoplankton community in the southeast Atlantic Ocean showed no significant toxic response at a NM-300 K concentration that resulted in ~40% biomass loss in the culture studies, suggesting a modulating effect of natural seawaters on Ag toxicity.

## Introduction

The increasing inputs of nanoparticles (NPs) into the environment constitutes a widespread pollution pressure^1^. Silver NPs (AgNPs) in particular represent a dominant fraction of manufactured NPs because of their advantageous antibacterial, antiviral and antifungal properties^2^. Despite their enhanced release rates into the marine environment, relatively little is known about the toxic effects of AgNPs on phytoplankton (photosynthetic algae), which are the base of the marine food chain. Diatoms are a group of phytoplankton that are globally dominant in coastal waters^3^. Factors affecting their growth rate therefore exert a direct forcing on phytoplankton community structure, networks of biogeochemical cycles, and the production rate of organic material that is made available for higher tropic levels (e.g., fisheries)^4^.

A number of studies have investigated the toxicity of AgNPs to marine and freshwater phytoplankton^5–11^. Toxicity of AgNPs to phytoplankton has been attributed to the release of Ag(I) from AgNPs, either within their growth medium or after uptake of the NPs into cells^12–16^. The general mechanism underlying toxicity of Ag ions themselves is relatively simple: numerous cations play fundamental roles in most cellular processes, and phytoplankton have evolved mechanisms to maximise uptake and storage of these metals^17^; however the inability, or partial ability, to discriminate between essential and non-essential cations (e.g. Ag(I)) leads to high levels of inadvertent incorporation of the incorrect metal, rendering proteins and associated metabolic pathways dysfunctional^17, 18^. However, a complication of this mechanism with regards to NPs is that toxicity of AgNPs has not always been found to be uniquely associated with the release of Ag(I)^9, 19–21^. For example, under exposure to AgNPs, elevated production of reactive oxygen species (ROS)^6, 22^, the reduction of light availability due to adsorption of NPs on the cell surface^6, 23^, and the enhanced presence of exopolymeric substances (EPS)^13, 24^ have all been suggested to contribute to toxicity beyond the influence of Ag ions alone.

An inherent complexity in interpreting the results of studies investigating the potential toxic effects of AgNPs on algae results from the diversity in behaviour of different types of NP added to the growth media. In particular, dynamic NP aggregation/oxidation processes occur, regulating NP interactions with algae^7, 10^. Any given sample of AgNPs has a set of distinct physicochemical properties specific to the particular medium they are suspended in, which has a strong dependence on the size of the NPs and their surface coating. For example, dominant factors controlling the aggregation, sedimentation, and dissolution of AgNPs in seawater are the ionic strength, pH, oxygen concentration, and the presence of organic matter or other Ag complexing agents such as sulfur species or organic thiols^25–28^.

Alongside physicochemical processes, characterisation of Ag speciation is critical for mechanistically linking AgNP dissolution to toxicity^29^. According to thermodynamic calculations, both sulfur and chloride silver species are the most common compounds in fresh and sea waters^28^. Transformation of pristine AgNPs prior to entering into natural waters (e.g., in wastewater treatment plants), or while present in the environment, is likely to occur. These transformations should not be overlooked as they can severely affect NP’s bioavailability. For example, the sufidation of Ag/AgNPs to form Ag_2_S, mainly found in sewage sludge, is a known process in wastewater treatment, which makes AgNPs less bioavailable^30^. However, according to Eh-pH diagrams, in the dual presence of sulfur and chloride at concentrations typical of seawater, chloride-silver species are predicted to be favoured in oxygenated waters; while under anoxic conditions sulfur-silver compounds predominate^28^. Therefore, oxidation of any Ag_2_S species produced during wastewater treatment processes to AgCl_x_ species in natural aerobic seawaters is expected. The dominant silver species formed in seawater are the anionic chlorocomplexes AgCl_2_^−^, AgCl_3_^−2^ and AgCl_4_^−3^, which have an opposite charge to Ag^+^. Cysteine is a very strong Ag complexing agent that is typically present in natural waters at nanomolar to milimolar concentrations^31^. Formation of Ag-cysteine complexes is thought to prohibit phytoplankton uptake of Ag(I) ions^12^; However, the role of cysteine in the context of AgNPs is probably more complex, as whilst it can strongly bind and remobilise Ag(I), it can also affect the aggregation and dissolution of AgNPs by adsorption onto their surfaces^32, 33^. Such processes therefore potentially have the capacity to enhance or reduce toxicity.

Here we report the results of a comprehensive laboratory study assessing the response of the coastal dwelling centric diatom *Chaetoceros curvisetus* to the reference Ag nanomaterial NM-300K, which is used in studies testing, and evaluating the hazards and safety of NPs^34^. The NM-300K AgNPs were used as delivered from the manufacturer without further purification/modification in order to preserve their validity as a representative nanomaterial. We evaluated the time-resolved (up to 48 h) toxic effects of AgNO_3_ and AgNPs on phytoplankton biomass and physiological status and employed supplementary experiments to resolve the underlying mechanisms of toxicity. Specifically, toxicity tests were performed by exposing the phytoplankton to AgNO_3_ (positive control); NM-300K (AgNP + Ag(I) species); NM-300K retained within a dialysis membrane, and NM-300K + cysteine to distinguish between the relative roles of Ag(I) and AgNPs on toxicity. We evaluated the production of reactive oxygen species, usually associated to AgNP toxicity, measuring H_2_O_2_ in some experiments. In an initial attempt to translate our observations in the culture studies, we also tested the response of a natural phytoplankton assemblage community off the coast of Namibia (southeast Atlantic Ocean) to treatment with the same NM-300K AgNP solution.

## Results and Discussion

### AgNP characterization

The mean intensity-weighted hydrodynamic diameter (d_h_) of NM-300K AgNPs measured using DLS in high-purity water was 48 ± 4 nm (PDI 0.37 ± 0.10). The size evolution over time was also measured in the culture medium to check for possible NP aggregation during the toxicological experiments (up to 48 h). The obtained d_h_ value, 56 ± 5 nm (PDI 0.35 ± 0.10), was higher than that found in high-purity water due to the loss of primary AgNPs by formation of aggregates and showed little change over time (Supplementary Fig. S1). The zeta potential measured in high-purity water was −4 ± 1 mV. The obtained value itself, close to neutrality, does not explain the low tendency of NM-300K AgNPs to aggregate in high ionic strength solutions (e.g., in the culture medium). The observed stability is attributed to a large amount of adsorbed non-ionic surfactants that provide a strong steric stabilization^35, 36^.

The presence of AgNPs in solution attenuates light and consequently provides a distinctive surface plasmon resonance band (SPRB). This band is apparent at around 380–420 nm and is the result of photon absorption by surface electrons^37^. The SPRB peak height correlates with the concentration of AgNPs in solution. Changes in the SPRB, detected by UV-vis spectrophotometry, allowed us to quantify the removal of non-aggregated AgNPs from solution^27, 38^. The x-axis position of the SPRB maximum in high-purity water declined over time (up to 48 h) from 412 to 409 nm, while the width at half peak height value—corresponding to the size distribution of AgNPs—declined from 78 to 72 nm. A similar trend in the SPRB was observed for the AgNPs in the culture medium, but at higher values: a decline in the position of the SPRB maximum from 441 to 437 nm, and from 90 to 80 nm for the width at half peak (Supplementary Fig. S2). The small blue shift in the SPRB position over time, which occurs in both high-purity water and the culture medium, likely reflects modest AgNP dissolution, resulting in a reduction in NP size. The red shift in the SPRB of around 29 nm and the increase in the width at half peak, when AgNPs were dispersed in culture medium compared to that observed in high-purity water, indicates the loss of primary AgNPs by formation of aggregates^39^. The height of the SPRB in high-purity water was stable, while in culture medium showed certain decline over time (∼4 and 15% at 24 and 48 h, respectively), in agreement with the AgNPs dissolution observed in this study and that of Zook *et al*.^40^.

The role of cysteine in the behaviour of AgNPs is complex. Different cysteine:Ag ratios, AgNPs coatings, and solution media, could all potentially produce different effects on AgNP aggregation and dissolution processes, with implications for the bioavailability of Ag species. There are a range of examples in the literature showing different behaviours of AgNPs in presence of cysteine: both increases and decreases in the amount of dissolved Ag released from AgNPs^41, 42^, AgNP surface modification^43^, AgNP aggregation^41^, and even contradictory effects including both AgNP disintegration and formation^44^. Luoma *et al*. and Gondikas *et al*. reported that AgNO_3_ and Ag(I) released from AgNPs can form dissolved species or complexes (polymers, aggregates, or particles) depending on the cysteine:Ag ratio^32, 45^. During our experiments there was no significant effect on the dissolution of AgNPs when cysteine was added to the culture medium.

Dissolved Ag(I) in the stock AgNP NM-300K solution was found to be less than 3% of the total Ag content without significant change over time (up to 48 h). Oxidation of AgNPs during our toxicity experiments showed significant variability depending on the AgNP concentration and time point. After 24 h the percentage of Ag(I) species in the test medium varied between 4.5 and 29% for 1000 and 10 ppb total Ag concentration, respectively. At 48 h the percentage of dissolved Ag increased to 25 and 92% for 1000 and 10 ppb total Ag, respectively.

We hypothesize that interactions of silver chloride species or cysteine with the surface of the NPs may play a key role in NM-300 aggregation/oxidation, despite its high stability in water containing an elevated chloride concentration (culture medium)^46, 47^. In addition, the presence of phytoplankton may also influence the dissolution/aggregation behaviour of AgNPs.

### Silver and cysteine speciation in the culture medium

The calculated speciation diagram for AgNO_3_ (0 to 10 µmol kg^−1^) in the culture medium suggested three dominant Ag species: AgCl_3_^−2^ (17.8%), AgCl_2_^−^ (22.7%) and AgCl_4_^−3^ (59.0%), with AgCl(aq) and free Ag^+^ ions representing <0.5%. During the toxicity experiments pH varied between 7.5 and 8.5 but the silver speciation remained roughly constant. The presence of cysteine in the culture medium caused preferential Ag(I) binding to the sulfur group of this organic thiol. According to speciation calculations at a cysteine: dissolved Ag(I) ratio of 10, the dominant compound was AgCys^−^, representing between 95 and 99.5% of the dissolved silver species at pH 7.5 and 8.5, respectively (neutral AgCys 4.20–0.44%; negligible silver chloride species). When silver was not present in the culture medium, ∼90% of the cysteine appears in solution, mainly as neutral H_2_Cys (60% at pH 7.5) or negatively charged HCys^−^ (65% at pH 8.5) (Supplementary Fig. S3). Cystine, the oxidised form of cysteine, is considered not to form complexes with Ag(I)^48, 49^.

### Effect of AgNPs on diatom biomass and physiological status

**Figure 1:**
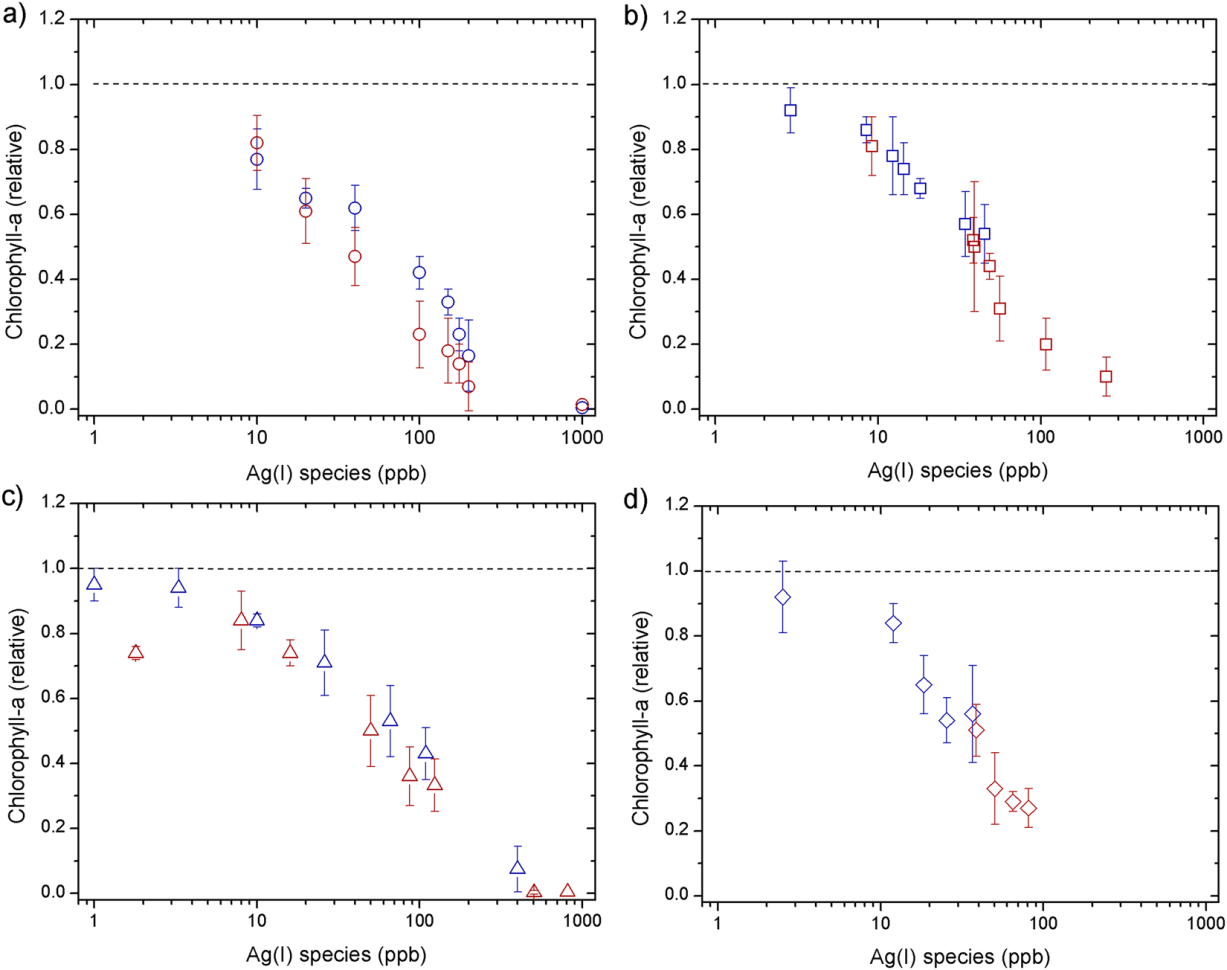
Responses of chlorophyll-a concentrations (normalized to control culture) measured at 24 h (blue) and 48 h (red) for toxicity experiments supplying: (**a**) AgNO_3_ (**b**) AgNP NM-300K, (**c**) AgNP NM-300K in dialysis bags and (**d**) AgNP NM-300K + cysteine. The symbols represent the average of 3–4 independent biological replicates (themselves an average of 2 technical replicates). The error bars represent the standard deviation of the biological replicates. Symbols with no error bars had one biological replicate only.

**Figure 2:**
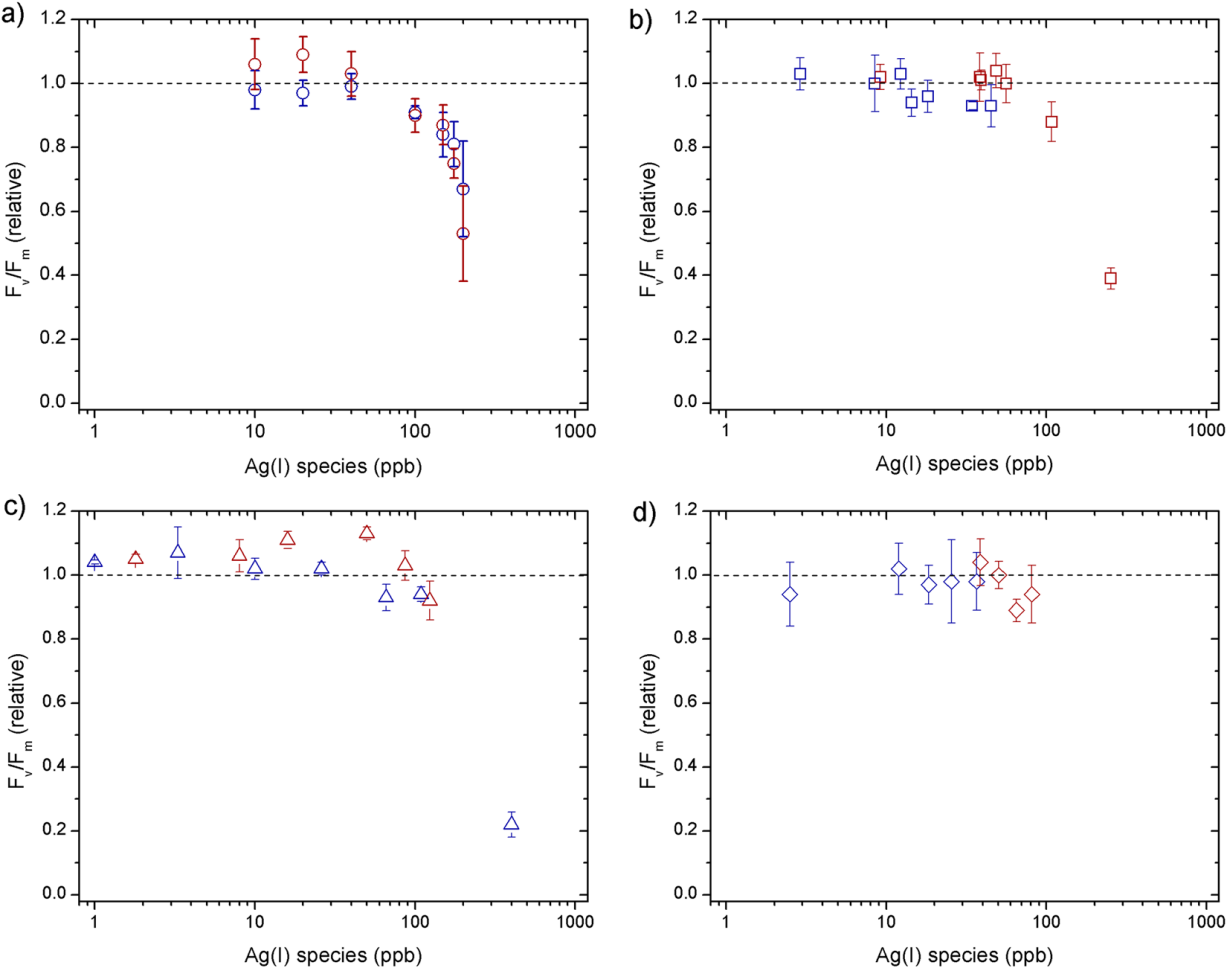
Responses of F_v_/F_m_ values (normalized to control culture) measured at 24 h (blue) and 48 h (red) for toxicity experiments supplying: (**a**) AgNO_3_, (**b**) AgNP NM-300K, (**c**) AgNP NM-300K in dialysis bags and (**d**) AgNP NM-300K + cysteine. The symbols represent the average of 3–4 independent replicates (2 repetitions each). The error bars represent the standard deviation of the measured replicates. Symbols with no error bars had one biological replicate only.

**Figure 3:**
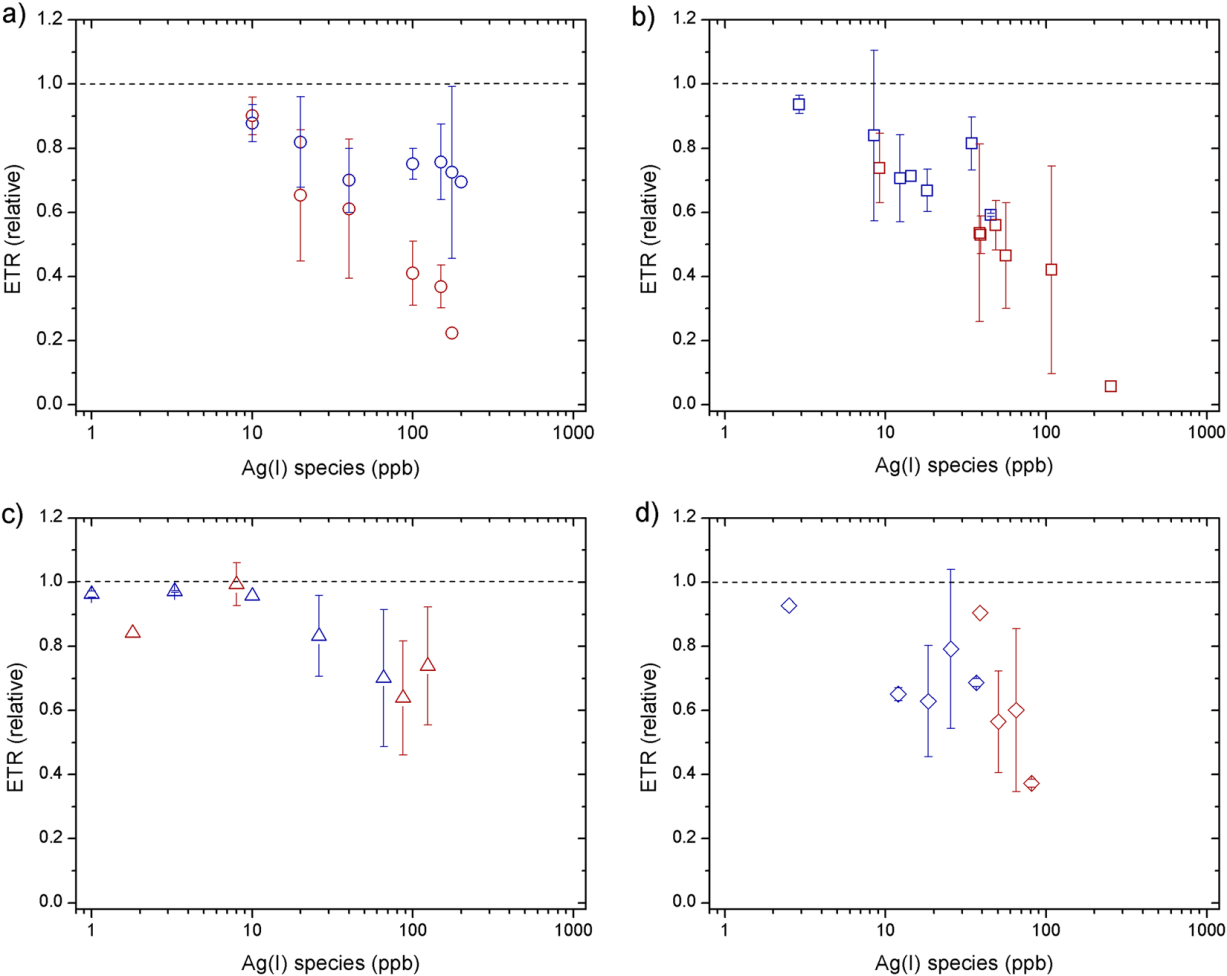
Responses of ETR_max_ values (normalized to control culture) measured at 24 h (blue) and 48 h (red) for toxicity experiments using: (**a**) AgNO_3_, (**b**) AgNP NM-300K, (**c**) AgNP NM-300K in dialysis bags and (**d**) AgNP NM-300K + cysteine. The symbols represent the average of 3–4 independent replicates (2 repetitions each). The error bars represent the standard deviation of the measured replicates. Symbols with no error bars had one biological replicate only.

Phytoplankton biomass declined relative to untreated controls at all supplied AgNO_3_ concentrations (Fig. 1a). The decline was exponential with supplied AgNO_3_, with near-complete cell death at 1000 ppb after 24 h. No clear time dependence was observed, with slight decreases in chlorophyll-a (as an indicator of biomass) from 24 to 48 h (Fig. 1a). F_v_/F_m_ (PSII photosynthetic efficiency) remained similar to the control at low Ag concentrations (<100 ppb), but rETR_max_ (relative maximum electron transport rate) showed clear decreases at Ag concentrations >10 ppb, which were more accentuated after 48 h (Figs 2–3a).

Once inside cells, potential causes of the reductions in both F_v_/F_m_ and rETR_max_ include Ag ions displacing essential trace metals in proteins of the photosynthetic apparatus, rendering them non-functional^18, 50^. Resulting modifications to electron flow through photosystems, as indicted by rETR_max_ changes, may then lead to intracellular ROS generation causing subsequent damage of the D1 protein, reflected in the reduced F_v_/F_m_ values at higher Ag ion concentrations^18, 51, 52^. Specifically, given similar binding capacities, Ag ions are thought to displace Cu, and therefore lead to dysfunction of Cu-containing plastocyanin and cytochrome oxidase^50^.

**Figure 4:**
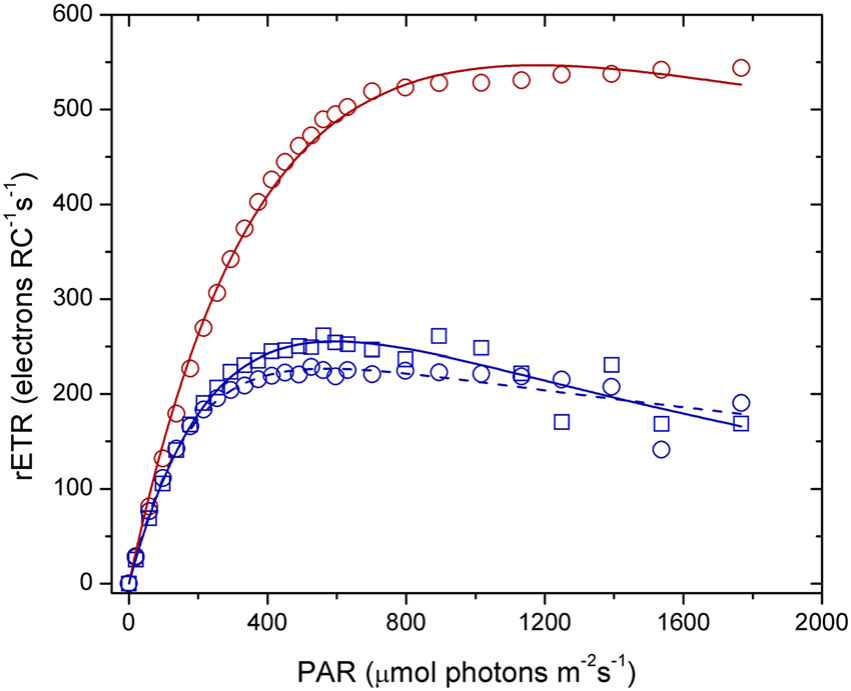
Electron transport rate per reaction centre (RC) for an example control (red circles), AgNO_3_ 100 ppb (blue circles, dashed line) and AgNP 400 ppb (∼108 ppb Ag(I)-species) (blue squares, solid line) in culture media after 48 h. The lines represent fits to the data using the photosynthesis-irradiance model of Platt *et al*.^61^.

A similar trend in chlorophyll-a biomass response as for AgNO_3_ was observed for the AgNPs at equivalent measured Ag(I)-chloride concentrations (Fig. 1b). F_v_/F_m_ showed little decline for all doses and time points apart from at the highest AgNP dose (∼250 ppb Ag(I)-chloride at 48 h; Fig. 2b). As for the AgNO_3_ experiments, declines in rETR_max_ were apparent at all Ag(I)-chloride concentrations >10 ppb (Figs 3b, 4).

As reported previously^6, 9, 11–13, 16^, our results suggest that AgNPs have the potential to severely impact growth of marine diatoms, with the magnitude of the toxic effect strongly modulated by the supplied NP concentration. A poorly resolved factor in previous experiments was the relative roles of the NP itself and the Ag(I) ions released by them in causing AgNP toxicity. To distinguish between these factors, at least for the specific AgNPs and diatom species used in this study, we separated the AgNP and diatoms using an ion permeable membrane. When NPs were placed in a dialysis bag (1000 kDa), which eliminated direct interaction of the NPs (≥ ∼10 nm) with diatom cells whilst allowing unrestricted passage of Ag ions, chlorophyll-a declined in a similar manner as for equivalent Ag doses for the AgNO_3_ experiment (Fig. 1c). Additionally, as for the AgNP experiment whereby diatoms were grown in culture medium in direct contact with AgNPs, F_v_/F_m_ remained high at Ag concentrations <100 ppb, whilst rETR_max_ was reduced (Figs 2–3c). These results suggest that the decline in chlorophyll-a in the AgNP treatments was not caused by mechanisms that have previously been invoked as driving mechanisms of toxicity: (i) uptake of the AgNPs themselves^20^, or (ii) interaction of the NPs with the cell surfaces^5, 6^. Moreover, measurements of H_2_O_2_ production—previously invoked as mechanism for AgNP toxicity via oxidative stress^6, 22^—at different AgNP and AgNO_3_ doses did not show any clear trend (Supplementary Fig. S4), e.g. increasing with Ag concentration, suggesting that this was not a major contributing factor for toxicity.

The remaining mechanism, uptake of Ag(I)-chloride ions themselves, should thus be identical between AgNO_3_ and AgNP treatments for a known NP release of Ag(I)-chloride. Accurate quantification of Ag(I) species released from NPs is difficult, hindering direct comparison of AgNO_3_ and AgNP treatments in this regard^53^. The clear similarity of chlorophyll-a biomass responses, albeit with an enhanced response lag time for NPs, alongside the observed similar changes in rETR_max_ and F_v_/F_m_, supports this conclusion. Our interpretation is therefore that the mechanism of toxicity is mostly via the release of Ag(I) ions from the NPs forming bioavailable Ag(I)-chloride species in the culture medium (section 2). There is a time lag between addition of the AgNPs and chlorophyll-a biomass response; additional Ag(I) release time (i.e. >24 h) and/or multiple agitation steps may be required to liberate sufficient Ag(I) to induce the chlorophyll-a decline. The aggregation of AgNPs showed little change over time (section 1), so it is expected not to have a significant contribution to toxicity. Nevertheless, it is worth noting that the presence of phytoplankton in the toxicity experiments could potentially modify the aggregation behaviour of AgNPs in the culture medium.

We calculated EC50 values using the declines in chlorophyll-a biomass with Ag^+^ dosage to indicate the toxicity end point (Table 1; Supplementary Fig. S5a–d). Across all Ag treatments and times points, EC50 ranged between 32–81 ppb, which is slightly lower than the previous published estimates for marine diatoms of 156 ppb AgNO_3_ (*S. costatum* ^6^) and ~100 ppb total Ag^+^ from supplied AgNPs (*T. weissflogii* ^13^). Moreover, all of our EC50 values fall within that of the range measured for aquatic and terrestrial organisms and mammalian cells treated with the NM-300K reference material, as recently compiled by Köser *et al*.^36^.

**Table 1:** Ag(I) concentration that decrease chlorophyll-a by 50% (EC50, ppb) for the phytoplankton *Chaetoceros curvisetus*.

	AgNO_3_	AgNP	AgNP + Cysteine	AgNP + Dialysis
24 h	68 ± 8	46 ± 7	36 ± 8	81 ± 9
48 h	42 ± 6	41 ± 9	32 ± 19	63 ± 10

More generally, declines in both chlorophyll-a and Fv/Fm values with increasing supplied Ag(I) concentrations, as observed here, have been reported in several previous phytoplankton toxicity studies^6, 12, 13^. In these studies, variable AgNP/AgNO_3_ doses were needed to result in equivalent impacts on phytoplankton biomass and physiological status, however they use different AgNPs and phytoplankton species.

In direct contrast to previous studies^12, 13^, the supplied cysteine appeared not to effectively modulate phytoplankton uptake of Ag(I), as similar biomass loss and rETR_max_ reductions were observed for given supplied Ag concentrations with or without cysteine (Figs 1b,d and 3b,d). Moreover, the lowest EC50 values were actually obtained when cysteine was present in solution (Table 1). We performed tests that confirmed the stability of cysteine in the culture media (see Supplementary Information and Fig. S6), which leads us to hypothesise that soluble cysteine-bound Ag species are readily taken up by this diatom^45^. Considering that diverse mechanisms exist for oceanic phytoplankton to access strongly bound metals (e.g. Fe, Zn, Co, Cu) that are often at bio-limiting concentrations in seawater, this should perhaps not be surprising^54^. This finding is of potential importance as it suggests the dual presence of Ag and cysteine in seawater does not preclude toxicity to some phytoplankton species.

Our culture experiments provided strong evidence for AgNP toxicity under laboratory conditions; in order to test if these findings translated to natural marine phytoplankton communities, we conducted bioassay treatments experiments in moderately productive (0.75 mg chlorophyll-a m^−3^), haptophyte-dominated seawaters in the southeast Atlantic. AgNPs were supplied at 40 ppb Ag(I), a concentration that resulted in ~40% loss of phytoplankton biomass in the laboratory experiments (Fig. 1b). In direct contrast to the laboratory experiments we observed no significant difference in chlorophyll-a or F_v_/F_m_ between control and AgNP-spiked samples after 48 h incubation (Supplementary Fig. S7). The apparent complete lack of toxicity imposed by the AgNP additions in this natural system either suggests that Ag ions released from AgNPs were effectively rendered non-bioavailable (for example due to complexation by organic matter—natural or purposely produced by the microbial community) or simply were not toxic to the specific phytoplankton assemblage encountered. Regardless, this finding highlights that additional complexity in natural systems must not be overlooked when translating results of such culture studies to the natural environment.

## Conclusions

We found exposure of diatom cultures to AgNPs resulted in rapid declines in biomass, maximum relative electron transport rates, optimum light intensities, and an increased susceptibility to photoinhibition. Retaining NPs in dialysis bags resulted in similar levels of toxicity as direct exposure to AgNPs, pointing towards a direct role of Ag ions in the extracellular growth medium in dictating the toxic responses. Intriguingly, and in direct opposite to previous studies^12, 13^, we found that addition of cysteine alongside Ag only marginally moderated its toxic effect. We hypothesise Ag-cysteine complexes are bioavailable to the phytoplankton species tested in this study. In contrast to our laboratory findings, the toxic response of a natural phytoplankton assemblage community off the coast of Namibia (southeast Atlantic Ocean) to exposure to the same AgNP solution was insignificant, suggesting additional complexity when transferring the results of laboratory toxicity studies to natural systems.

## Methods

### Nanoparticles

Experiments were conducted using NM-300K nano-Silver <20 nm reference nanomaterial from the Fraunhofer Institute for Molecular Biology and Applied Ecology (Germany). The NM-300K sample dispersion is yellow-brown in colour and has a nominal silver content of 10% (w/w). The NPs are stabilized with 4% (w/w) of the surfactant Polyoxyethylene Glycerol Trioleate and Polyoxyethylene (20) Sorbitan mono-Laurat (Tween 20). According to the manufacturer, the AgNPs in the stock solution are spherical and have a transmission electron microscope (TEM) measured diameter of 15–20 nm. The NM-300K stock solution used in the toxicity test was prepared according to The European Commission Joint Research Centre (JRC) recommendations and stored refrigerated^34^. Freshly prepared dilutions of the NM-300K stock were used for each set of experiments, which collectively lasted for a duration of three months.

The size distribution of the AgNPs was obtained with Dynamic Light Scattering (DLS) measurements using a Malvern Zetasizer Nano ZS following termination of toxicity experiments. The size evolution over time of the NM-300K AgNPs (1.1 mgL^−1^) was determined in high-purity water (18.2 MΩ cm^−1^, MilliQ, Millipore) and culture media (see below for details of the culture media). Electrophoretic mobility values were used to calculate the surface charge (zeta potential) of the studied NPs in high-purity water using Henry’s equation under the Smoluchowski’s approximation within Malvern software^55^. The surface plasmon resonance band (SPRB), a characteristic signal of AgNPs in the UV-visible spectra, was analysed using a deuterium tungsten halogen light source (DT-Mini 2GS, Ocean Optics), a quartz cuvette and a miniature CCD array spectrophotometer (USB-4000, Ocean Optics), connected through two optical fibres (600 μm fibre, P600- 025-SR).

### Phytoplankton culture conditions

The diatom *Chaetoceros curvisetus* was grown in 250 mL sterilized culture flasks under a 12 h day-night cycle. Supplied irradiance was spectrally neutral (550 µmol photons m^−2^ s^−1^) and temperature was maintained at 18 °C. The phytoplankton were grown in slightly modified Aquil culture media prepared from filtered seawater^56^ until the exponential growth phase was reached (see Supplementary Information for details), whereupon 10 mL was sub-cultured into 100 mL of freshly prepared culture media at experimental start points.

### Toxicity experiments

Several Ag treatments were carried out in separate experiments: (1) AgNP; (2) AgNO_3_ (i.e., free Ag ions); (3) AgNPs retained within a dialysis membrane (Spectra/Por^®^, Molecular Weight Cut-Off: 1000 kDa); (4) AgNPs + cysteine (total silver equimolar concentration) to assess for potential modulating effects of the complexation of Ag ions by an organic sulfur compound on toxicity. Each experiment was run at several total Ag concentrations ranging between 10–1000 ppb (µgL^−1^). Two controls, where phytoplankton was grown in untreated culture medium, were conducted alongside each of the four Ag treatments. Culture flasks were manually agitated 3 times per day to limit settling of AgNPs and phytoplankton. Each experiment was subsampled at 24 and 48 h before the light cycle started (i.e., representing dark acclimated phytoplankton) for quantification of chlorophyll-a as an indicator of biomass, the physiological state of phytoplankton, alongside samples for Ag analysis.

A bioassay experiment was conducted in the southeast Atlantic (15.04°E, 27.97°S) to test the response of a natural marine phytoplankton community to addition of AgNPs^57^. Briefly, 1 L acid-washed Nalgene polycarbonate bottles were filled with surface seawater (~3 m depth) on a research cruise in December 2015 using a sampling device free from contamination by trace metals. Three samples were amended with AgNPs, three with AgNPs + 2 nmol L^−1^ Fe^2+^, and three bottle bottles were sealed with no amendment. The purpose of the NP + Fe treatment was to assess for diminished toxicity when a cation micronutrient, potentially competing with Ag^+^ ions for cellular uptake, was added in excess of typical seawater concentrations. Bottles were incubated in on-deck incubators for 48 h. Irradiance in the incubators was regulated to sea surface conditions using blue screening (Lee filters, ‘blue lagoon’) and temperature was regulated by flushing with continuously flowing surface seawater pumped directly onto the ship. After 48 h the incubated samples were analysed for chlorophyll-a concentrations and photophysiological condition (see below).

It should be appreciated that the range of AgNP concentrations used in this work (ppb) is higher than the future predicted concentrations in natural waters (ppt). As such, whilst effective in illustrating thresholds and mechanisms of toxicity, direct extrapolation of obtained results to the lower concentrations projected in the future should be undertaken with caution.

### Chlorophyll-a concentrations and calculation of EC50

Sub-samples of 1 mL were pipetted from each culture flask after gentle agitation and filtered through a 25 mm GF/F filter (nominal pore size of 0.7 µm). Filters were folded in half and placed in centrifuge tubes with 10 mL of 90% acetone that were then ultrasonicated for 4 min and stored in the dark at −20 °C for at least 24 h before analysis. Chlorophyll-a concentrations were measured at a constant temperature (18 °C) using a blank-corrected fluorescence spectrophotometer (Varian Cary Eclipse) before and after acidification following the method of Knap *et al*.^58^ (see Supplementary Information). The effective silver concentration resulting in a 50% reduction in chlorophyll-a (EC50) was calculated by fitting the chlorophyll-a response curves to an exponential decay function (y = A e^−x/t^), where A and t are parameters optimised by the nonlinear least squares minimization tool of the software OriginPro 8, based on the Levenberg–Marquardt algorithm. Errors on A and t, reflecting fit quality and variability in the 3–4 replicates at each concentration, were calculated during the software’s fitting routine and used to calculate reported EC50 error ranges.

### Phytoplankton physiological state

A Fast Repetition Rate fluorometer (FRRf, FastOcean) equipped with a FastAct laboratory system (both Chelsea Technologies Group) was used to measure the apparent PSII photosynthetic efficiency (F_v_/F_m_) and generate curves of relative electron transport rate (rETR) versus irradiance. The FRRf supplied 100 × 1 µs excitation light flashes separated by 1 µs followed by 40 × 1 µs relaxation flashes separated by 50 µs. Fluorescence transients were fit to the model of Kolber *et al*.^59^ within Fastpro8 software provided by the manufacturer. The rETR values were calculated following Gorbunov *et al*.^60^ and the photosynthesis-irradiance model of Platt *et al*.^61^ was fit to the data to obtain values of the maximum electron transport rate (rETR_max_), the light limited slope (α) and the photoinhibition parameter (β).

### Silver measurements

Silver concentrations were measured by ICP-MS (Quadrupole Thermo X-Series 2) after dilution of the samples with 0.3 mol L^−1^ HNO_3_ (70% Optima, Fisher Scientific). The potential release of Ag(I) from AgNPs was measured following the same protocol after ultrafiltration of the samples using two methods: centrifugal ultrafilters (3 kDa, Amicon Millipore), and centrifugation at 14000 rpm for 80 min (5430 R, Eppendorf).

Total Ag and Ag(I) were measured after 24 and 48 h in the four experiments for all the concentrations tested.

### H_2_O_2_ measurements

H_2_O_2_ concentration was measured by flow injection analysis using luminol chemiluminescence directly following the method of Hopwood *et al*.^62^. The detection limit was <1 nM, a linear chemiluminescence response (r^2^ = 0.994) from 3.8 to 460 nM was obtained, and Ag interference was accounted for.

### Silver and cysteine speciation in culture medium

Silver speciation was calculated using the software MINTEQ+^63^. Cysteine was included in MINTEQ + using the acid equilibrium constants (log K_a_ = 9.64, 17.64 and 19.14 for the formation of HCys^−^, H_2_Cys and H_3_Cys^+^, respectively) obtained in artificial seawater from Sharma *et al*.^64^. The formation constants for the silver-cysteine bond (log K_f_ = 21.54 and 15.2 for AgCys and AgCys^−^, respectively) were determined at I = 0.01 M by Adams and Kramer^65^.

### Data analysis and statistics

Three to four replicates of independent experiments (i.e. biological replicates) were carried out to obtain chlorophyll-a, Fv/Fm and rETRmax values. The sample measurements were normalized to an untreated control culture that was run in duplicate during each experiment. In Figs 1, 2 and 3 we show the average of the 3–4 biological replicates (themselves each a mean of 2 technical replicates) normalized to the mean of the control cultures. The error bars represent the standard deviation of the 3–4 biological replicates specific to the test. In a few cases, only 1–2 replicates could be measured (symbols reported with no error bar), for example due to problems arising when measuring signals below detection limit at the highest imposed Ag(NP) concentrations. Microsoft® Excel® 2011 was used for calculations of mean averages and standard deviations. OriginPro 8 was used for curve fitting procedures to derive EC50 calculations and plotting the Figures. Curve fitting for the rETR versus irradiance data and testing for statistical significance of responses in the field incubation experiments was conducted in R.

### Data availability

The datasets generated during and/or analysed during the current study are available from the corresponding author on reasonable request.

## Electronic supplementary material

Supplementary information
