# Supplementary material for: Mechanisms of silver nanoparticle toxicity to the coastal marine diatom Chaetoceros curvisetus
Source: Sci Rep. 2017 Sep 7;7:10777. doi: 10.1038/s41598-017-11402-x (PMC5589759; doi:10.1038/s41598-017-11402-x)
Supplement: Supplementary file 1 — Supplementary information [file 41598_2017_11402_MOESM1_ESM.pdf]

Supplementary Information for the research paper:

Mechanisms of silver nanoparticle toxicity to the coastal marine diatom Chaetoceros curvisetus

Pablo Lodeiro[a,b*], Thomas J. Browning[b], Eric P. Achterberg[a,b], Aurélie Guillou[b] and Mohammad S. El-Shahawi[c,1]

[a]Ocean and Earth Science, University of Southampton, National Oceanography Centre, European Way, SO14 3ZH Southampton, UK.

[b]GEOMAR Helmholtz Centre for Ocean Research Kiel, Wischhofstraße 1-3, 24148 Kiel, Germany

[c]Department of Chemistry, Faculty of Science, Damietta University, Damietta, Egypt.

*Corresponding Author: plodeiro@geomar.de

Present Addresses:

[1]Department of Chemistry, Faculty of Science, King Abdulaziz University, P. O. Box 80203, Jeddah 21589, Kingdom of Saudi Arabia (on sabbatical leave)

Culture medium preparation

The culture medium used during the toxicological experiments was prepared under clean conditions and stored refrigerated until used. The main compounds and their concentrations, selected based on previous culturing studies, are the following:

- Aged seawater collected from the Pacific (open ocean);
- Added compounds:

Nutrients: $NaNO_3$ 150 µmol L^{-1}; KH_2PO_4 10 µmol L^{-1}; $Na_2SiO_3{\cdot}9H_2O$ 100 µmol L^{-1}; Vitamins: Thiamin $2{\times}10^{-4}$ gL^{-1}, Biotin $2.2{\times}10^{-7}$ gL^{-1}, B12 1×10-6 gL^{-1}; Trace metals + EDTA: $Na_2EDTA{\cdot}2H_2O$ 100 µmol L^{-1}, $CuSO_4{\cdot}5H_2O$ 20 nmol L^{-1}, $Na_2MoO_4{\cdot}2H_2O$

100 nmol L^{-1}, $CuCl_2 \cdot 6H_2O$ 50 nmol L^{-1}, $MnCl_2 \cdot 4H_2O$ 115 nmol L^{-1}, $ZnSO_4 \cdot 7H_2O$ 80 nmol L^{-1}, $Na_2SeO_3 \cdot 4H_2O$ 10 nmol L^{-1}; Iron + EDTA: $Na_2EDTA \cdot 2H2O$ 1.5 µmol L^{-1}, $FeCl_3$ 1.5 µmol L^{-1}.

We filtered the solution with a pre-sterilized vacuum driven disposable filtration system (0.22 µm Millipore Express) before using it in the experiments. We refer to this filtered solution as the culture medium.

Chlorophyll-a measurements

A 100 mL standard solution containing approximately 0.96 mg of pure chlorophyll-a in 90% acetone was prepared. The accurate concentration of this solution was determined spectrophotometrically. The UV-visible spectrum of the chlorophyll-a solution was recorded using a light source (DT-Mini 2GS), a 1 cm cuvette and a miniature spectrophotometer (USB-4000, Ocean Optics), connected through two optical fibres (600 µm fiber, P600-025-SR). This method allowed us to measure the absorbance of the solution at a particular wavelength. Chlorophyll-a has two absorption maxima, one at 432 nm and another at 664 nm. The chlorophyll-a concentration was calculated as follows [1]:

$$chlorophyll - a = \frac{(A_{max} - A_{750nm})}{E \times l}$$

A_{max} : absorption maximum at 664 nm.

A_{750nm} : absorbance at 750 nm to correct for light scattering

E : chlorophyll-a extinction coefficient in 90% acetone at 664 nm (87.67 L g^{-1} cm^{-1})

l = cuvette path length (cm)

This standard chlorophyll-a solution was used to prepare calibration curves (from 0.6 to 34 µg L^{-1}). The obtained parameters from the calibration were used to calculate chlorophyll-a and phaeopigment concentrations in the samples:

$$chlorophyll - a = \frac{F_m}{F_m - 1} \times (F_0 - F_a) \times K_x \times \frac{V_{ext}}{V_s}$$

F_m: acidification coefficient (F/F_a)

F: reading before acidification

F_a: reading after acidification

K_x: door factor from calibration curves

V_{ext}: extraction volume

V_s: sample volume

Cysteine stability measurements

The stability of the cysteine solution used in the experiments was evaluated using a modified fluorometric method used for ammonium measurements [2]. Equal volumes of cysteine 1 mM and an OPA (o-phthaldialdehyde) amine-detection reagent were mixed together. The reagent was prepared mixing 100 mL of 0.3 M OPA/EtOH, 2 L of $Na_2B_4O_7$ 0.1 mol $L^{-1}$and 125 mL of Na_2SO_3 0.065 mol L^{-1} solutions. The florescence emission spectra of the solutions were recorded at fixed excitation (380 nm) and constant temperature (18 °C) using a high-purity water blank-corrected fluorescence spectrophotometer (Varian Cary Eclipse). The fluorescence spectrum of a freshly prepared cysteine solution was compared the spectrum of an identical solution aged for 4 months at 4 ºC (both prepared using pre-boiled high-purity water under N_2 flow and kept in dark).

As we did not perform time-resolved measurements of cysteine concentrations in the toxicity experiments themselves, we cannot completely rule out the possibility that instability of the cysteine in the culture medium resulted in limited Ag(I) ion complexation [3,4]. However, although unstable under oxic conditions [5,6], it is well known that cysteine does not readily undergo oxidation when stabilized by metals,

such as Ag [7], and we could find no evidence for cysteine degradation during any previous toxicity studies using AgNPs. Cystine, the oxidized form of cysteine, presents a double sulfur bound which does not form Ag(I) complexes [8]. In order to test this more rigorously, we tested possible oxidation of the cysteine used to spike the samples. We found no significant differences in the fluorescence spectra for a freshly prepared cysteine solution (1 mmol L^{-1}) compared to a solution aged for 4 months in the fridge, following addition of OPA reagent to both (Figure S5). Conversely, a clear suppression of the fluorescence signal was observed when the cysteine solutions were spiked with an oxidising agent (15% H_2O_2), confirming that cystine was not contributing to the fluorescent signal in the aged cysteine solution.

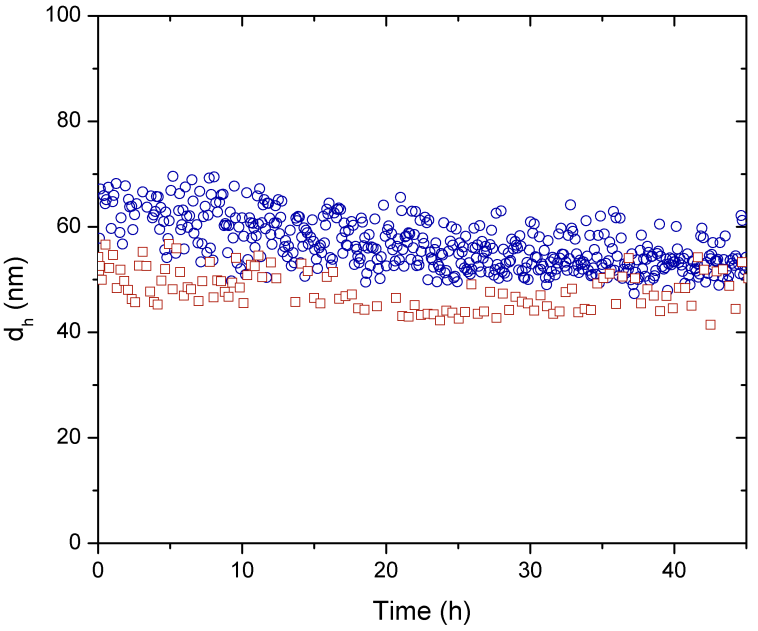

Figure S1. Hydrodynamic diameter (d_h) evolution over time of AgNPs NM-300K (1.1 ppm) measured in high-purity water (red squares) and culture media (blue circles).

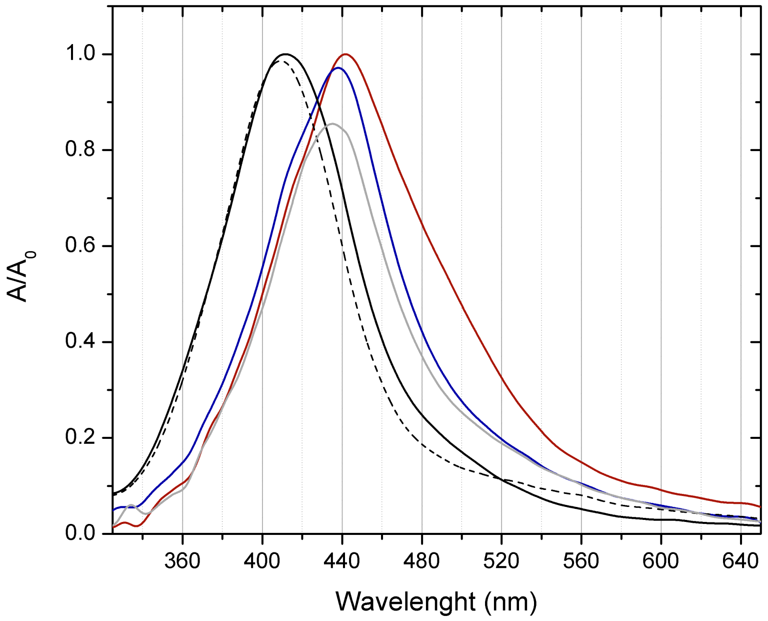

Figure S2. Normalized UV-Visible spectra of AgNP NM-300K (1.1 ppm) in high-purity water (black lines) and culture media, at different times: 0 h (black and red lines), 24 h (blue line), 48 h (dashed-black and grey lines).

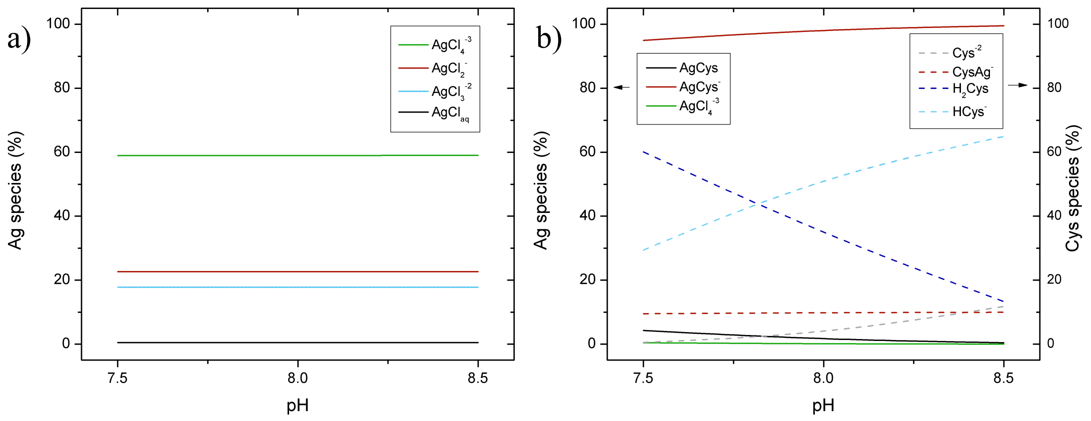

Figure S3. a) Silver speciation calculation obtained from MINTEQ+ as a function of pH in culture media for a total initial concentration of Ag(I) of 0.5 µmol L^{-1}.

b) Silver and cysteine speciation calculations obtained from MINTEQ+ (acid equilibrium and silver formation constants for cysteine were included) as a function of pH in culture media. Total initial concentration of Ag(I) and cysteine of 0.5 and 5 µmol L^{-1}, respectively.

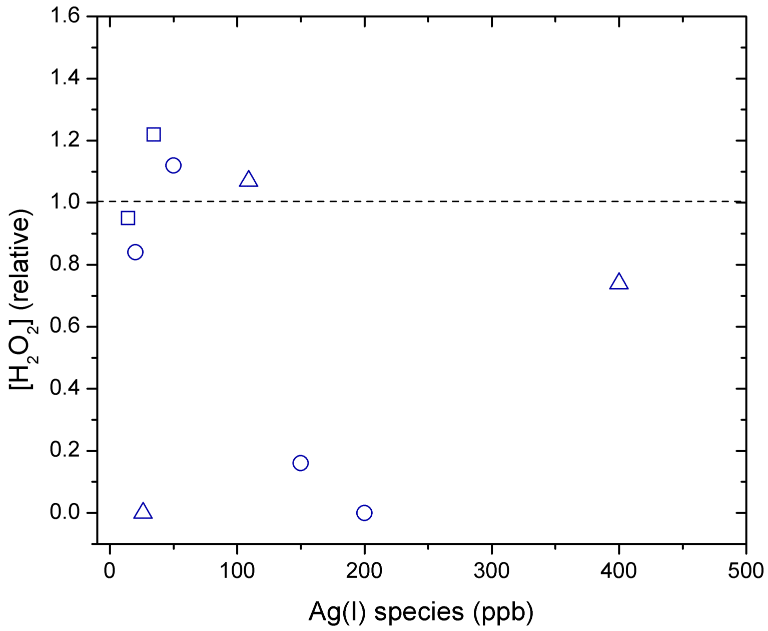

Figure S4. Control-normalized H_2O_2 concentration measured in culture media containing $AgNO_3$ (circles), AgNP NM-300K (squares) and AgNP NM-300K in a dialysis bag (triangles) after 24 h.

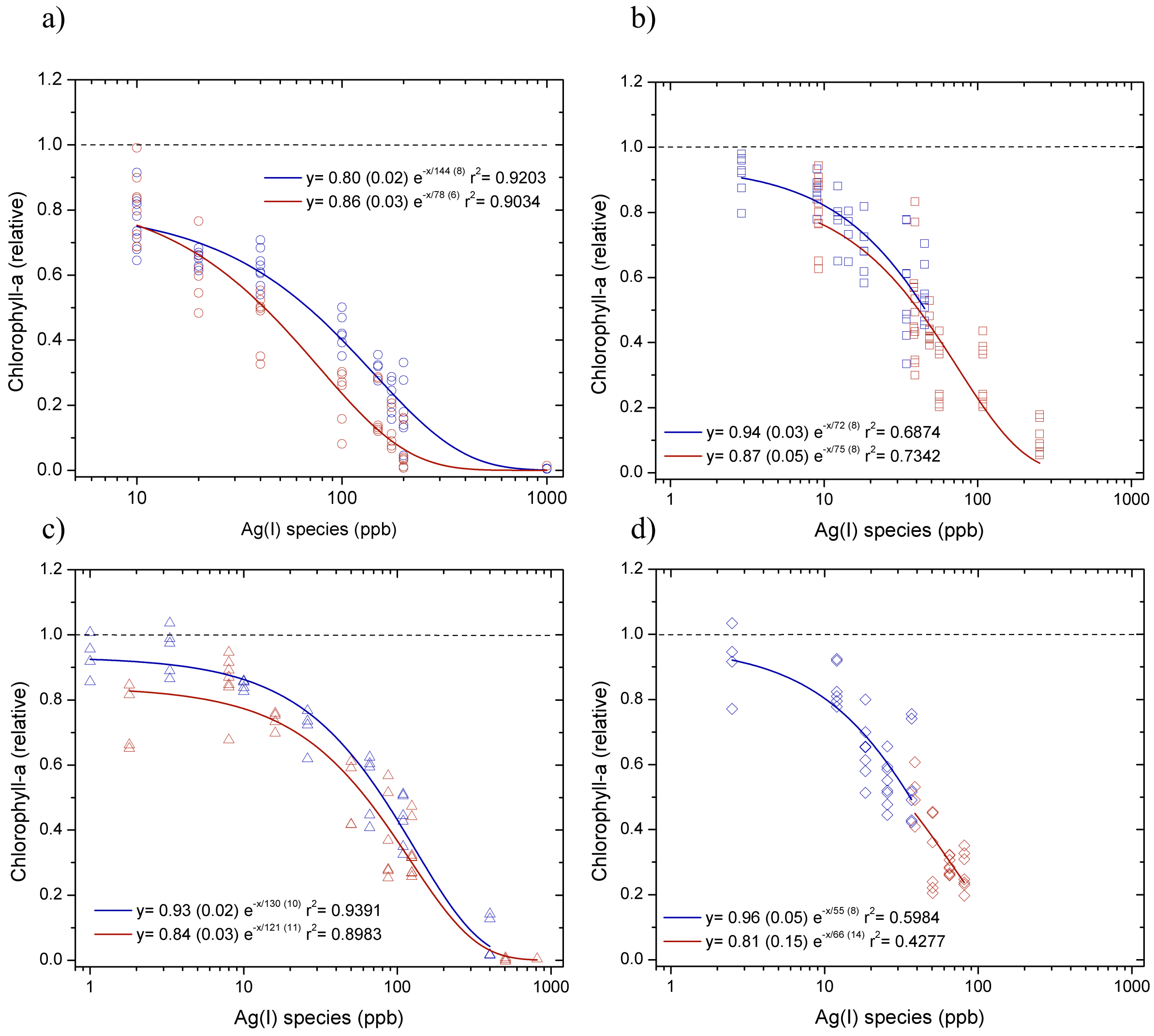

Figure S5. Responses of chlorophyll-a concentrations (normalized to control culture) measured at 24 h (blue) and 48 h (red) for toxicity experiments supplying: a) AgNO3 b) AgNP NM-300K, c) AgNP NM-300K in dialysis bags and d) AgNP NM-300K + cysteine. The symbols represent the data measured for all biological replicates (average and standard deviations are shown in Figure 1). The lines represent fit of the exponential decay function shown in the legend to the data at 24 h (blue line) and 48 h (red line). The fits were used to calculate EC50 (i.e., x value when y= 0.5).

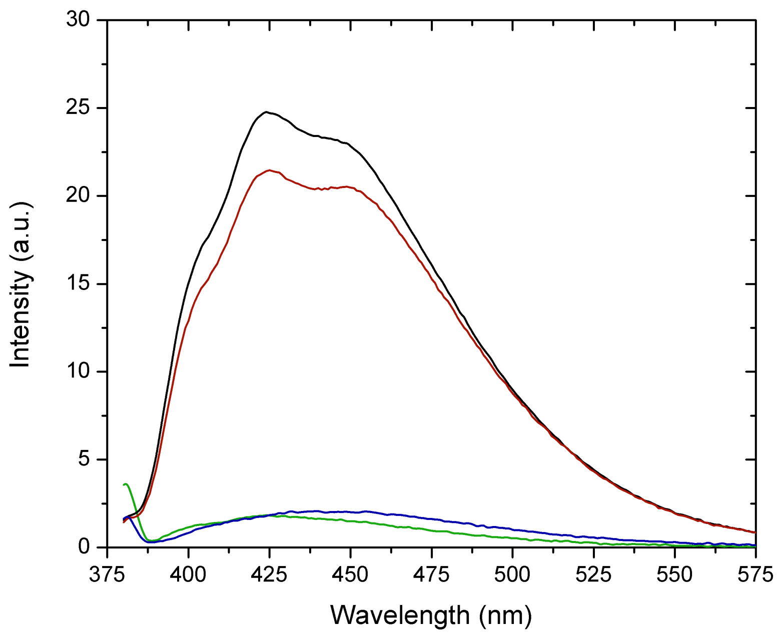

Figure S6. Fluorescence spectrum at fixed excitation (380 nm) of a freshly prepared 1 mmol L^{-1} cysteine solution (black line); an identical solution aged for 4 months at 4 ºC (red line); freshly prepared 1 mmol L^{-1} cysteine solution after spiking with 15% H_2O_2 (blue line) and control (green line).

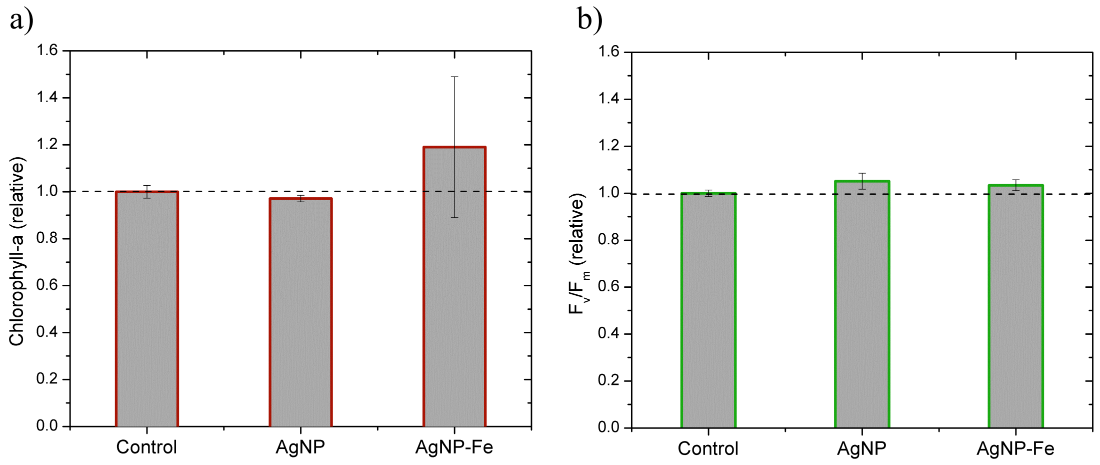

Figure S7. Responses of control-normalised chlorophyll-a concentrations (a) and F_v/F_m values (b) measured in the bioassay experiment conducted in the SE Atlantic after 48 hours incubation. The AgNP samples were amended with 40 ppb total Ag. AgNP-Fe indicates 2 nmol L^{-1} $FeCl_3$ was added in addition to the AgNPs. Bar heights represent the mean average of triplicate bottle incubations and error bars show the standard errors. Differences in means across treatments were statistically insignificant (ANOVA and Fisher PLSD test p<0.05).

References

1 Knap, A. H., Michaels, A., Close, A. R., Ducklow, H. & Dickson, A. G. Protocols for the Joint Global Ocean Flux Study (JGOFS) Core Measurements. *JGOFS, Reprint of the IOC Manuals and Guides No. 29, UNESCO 1994* **19**, 218 (1996).

2 Holmes, R. M., Aminot, A., Kérouel, R., Hooker, B. A. & Peterson, B. J. A simple and precise method for measuring ammonium in marine and freshwater ecosystems. *Can J Fish Aquat Sci* **56**, 1801-1808, doi:http://dx.doi.org/10.1139/f99-128 (1999).

3 Siriwardana, K. *et al.* Contradictory Dual Effects: Organothiols Can Induce Both Silver Nanoparticle Disintegration and Formation under Ambient Conditions. *J Phys Chem C* **119**, 20975-20984, doi:http://dx.doi.org/10.1021/acs.jpcc.5b05965 (2015).

4 Levard, C., Hotze, E. M., Lowry, G. V. & Brown, G. E. Environmental Transformations of Silver Nanoparticles: Impact on Stability and Toxicity. *Environ Sci Technol* **46**, 6900-6914, doi:http://dx.doi.org/10.1021/es2037405 (2012).

5 McBean, G. J. in *Biomolecular and Biomedical Science Research Collection* (ed F.V. and Karataev Chorkina, A.I.) (Nova Publishers, 2012).

6 Gondikas, A. P. *et al.* Cysteine-Induced Modifications of Zero-valent Silver Nanomaterials: Implications for Particle Surface Chemistry, Aggregation, Dissolution, and Silver Speciation. *Environ Sci Technol* **46**, 7037-7045, doi:http://dx.doi.org/10.1021/es3001757 (2012).

7 Hsu-Kim, H. Stability of Metal−Glutathione Complexes during Oxidation by Hydrogen Peroxide and Cu(II)-Catalysis. *Environ Sci Technol* **41**, 2338-2342, doi:http://dx.doi.org/10.1021/es062269+ (2007).

8 Bell, R. A. & Kramer, J. R. Structural chemistry and geochemistry of silver-sulfur compounds: Critical review. *Environ Toxicol Chem* **18**, 9-22, doi:http://dx.doi.org/10.1002/etc.5620180103 (1999).
